# Hypomethylation of *GCNT2* isoform A correlates with transcriptional expression and is associated with poor survival in acute myeloid leukemia

**DOI:** 10.3389/fimmu.2025.1490330

**Published:** 2025-02-17

**Authors:** De-hong Wu, Hong-chun Qiu, Jing Xu, Jiang Lin, Jun Qian

**Affiliations:** ^1^ Deparrtment of Central Lab, Affiliated People’s Hospital of Jiangsu University, Zhenjiang, Jiangsu, China; ^2^ Deparrtment of Hematology, KunShan Third People’s Hospital, Kunshan, Jiangsu, China

**Keywords:** *GCNT2*, DNA methylation, acute myeloid leukemia, stemness scores, survival, immunotherapy

## Abstract

**Background:**

The function of *GCNT2* has been documented to act as an oncogenic driver or tumor suppressor in different types of tumor, but the role of *GCNT2* and the epigenetic regulation mechanism in AML, however, has not yet been clarified. This study aimed to assay the expression and methylation profile of *GCNT2* in AML, and further elucidate the clinical significance.

**Methods:**

Multiple datasets from the Gene Expression Omnibus (GEO) and The Cancer Genome Atlas projects (TCGA) were used to explore the expression and methylation profile of GCNT2 in normal hematopoiesis and AML. A pan-cancer analysis was performed to define the survival implications of GCNT2 across multiple cancers including AML. The relationships between GCNT2 expression/methylation and clinicopathologic features were investigated using a TCGA-AML dataset. Correlation analysis was performed to explore the relationship between transcriptional expression and DNA methylation. Differentially expressed genes (DEGs) on the KEGG pathway and GO terms were visualized using DAVID. Gene Set Enrichment Analysis (GESA) was carried out to assess the underlying mechanism. The relationship between methylation and immune cell infiltration was also examined.

**Results:**

*GCNT2* expression was highest in hematopoietic stem cells (HSC) but gradually decreased during the hematopoiesis differentiation, the monocytes, however, remained a high level of *GCNT2* as an exception. In AML, *GCNT2* was down-regulated as compared to normal hematopoiesis but was much higher in contrast to normal peripheral blood samples. Data from a pan-cancer analysis revealed that high-expressed *GCNT2* contributed to a worse OS for AML. DNA methylation of *GCNT2* showed a distinctive co-methylation pattern in AML and significantly negatively correlated with transcriptional expression. Methylation in the transcriptional start site of isoform A plays a critical role in the epigenetic regulation of *GCNT2* expression. The silence of *GCNT2* in AML was attributed to DNA methylation. Hypomethylation of isoform A significantly predicted poor survival in AML, linking to several cytogenetic and molecular abnormalities, such as t (8:21), inv (16), t (15;17), and genes mutations of *DNMT3A*, *CEBPA*, *RUNX1*, and *WT1*. Enrichment analysis disclosed that hypomethylation of isoform A was involved in the immune system, and it was further revealed that hypomethylation of isoform A was tightly associated with immune cell infiltration and could be served as a promising indicator for immunotherapy.

**Conclusions:**

Our comprehensive research demonstrated that *GCNT2* acted as an oncogene in AML, and was epigenetically regulated by DNA methylation in isoform A. Hypomethylation of isoform A could be served as a promising indicator to identify the high-risk AML patients who might be responsive to immunotherapy.

## Introduction

Acute myeloid leukemia (AML) is a group of hematopoietic malignancies characterized by uncontrolled myeloid progenitor cells proliferating and accumulating in the bone marrow and circulation, leading to impairment of normal hemopoiesis and bone marrow microenvironment with the concomitance of cytopenia and immunodeficiency (1, 2). Intensive therapy, such as the “3 + 7” regimen induction, high-dose cytarabine, or allogeneic stem cell transplantation consolidation, remains the mainstay of treatment for the fit patient, resulting in a long-term cure rate of 30-40% (3, 4). Still, a large proportion of patients who are elderly or not eligible for intensive therapy, will succumb due to disease relapse or progression. It is believed that both genetic and epigenetic alterations profoundly participate in disease initiation, clonal evolution, drug resistance, disease progression, and recurrence (5, 6). Major efforts have been made to dissect the molecular abnormalities in AML diagnostic classification, prognostic prediction, targeted therapies, drug response, as well as the MRD monitor (7–11). In the past few years, AML treatment was essentially improved because of the advances in the understanding of the molecular or genetic mechanisms of the disease. The introductions of hypomethylating agents (HMAs), new targeted substances as well as immunotherapies substantially broadened the treatment options for AML. Particularly, the emerging approaches of immunotherapies such as antibody-based immune checkpoint blockade, bispecific antibodies, vaccines as well as cell therapies profoundly change the strategy of AML treatment, contributing to an enhancement of the therapeutic efficacy and an endurance of disease control. But due to the tremendous heterogeneity in genetics, cytogenetics, and epigenetics, only a small subset of patients with specific molecular alterations benefited from these treatments. More intensive research aimed at AML pathogenesis and progression is required, and it is unmet to explore more reliable biomarkers for risk stratification and treatment decision, and to develop more molecular targets for this disease.

Recently, ongoing research has focused on glycobiology in cancer. Fundamental changes of glycosylation have been observed in diverse cancers (12), and aberrant glycan modifications were shown to be associated with tumor cell growth, cellular communication, metastasis, invasion, as well as tumor immunomodulation, contributing to tumor initiation and development (13–16). Moreover, intensive studies have demonstrated aberrant glycosylation as a reliable biomarker and a promising target for tumor treatment (12, 17, 18). *GCNT2* refers to Glucosaminyl (N-Acetyl) Transferase 2, a gene locates in chromosome 6p24 and encodes an I-branching enzyme, which was initially reported to involve in the formation of blood group I antigen. The lost function of *GCNT2* due to the gene mutation was associated with adult i blood group phenotype as well as congenital cataracts (19–21). Later, emerging evidence revealed that the deregulation of *GCNT2* was implicated in various cancers (22–28). Upregulation of *GCNT2* was associated with poor prognosis in breast cancer, prostate cancer, and esophageal carcinoma, with the functions of promoting proliferation, migration, and invasion, acting as an oncogene (23, 24, 26), on the other hand, evidence from other studies supposed the tumor suppressive function of *GCNT2* in melanoma (25), endometrial carcinoma (29), and adult T-cell lymphoma/leukemia (28). Extensive research on melanoma has illustrated the opposite role of *GCNT2* in the pathogenesis of the disease, with loss of *GCNT2*/I-antigen contributing to metastasis and progress (25). The unique expression of *GCNT2* in melanoma has been explored for the prediction of survival and the therapeutic target (30). In addition to genetic alteration, epigenetic regulation, DNA methylation in particular, also contributes to *GCNT2* expression (31, 32), data from existing observations have supported a key role of aberrant methylation of *GCNT2* in several solid tumors, in which, hypomethylation of *GCNT2* was associated with tumor development, invasion, and distant metastasis (32–34), interestingly, hypomethylation of *GCNT2*, both in tumor tissues and even in the corresponding normal tissues, holds a promising capability in predicting lymph node metastasis and clinical outcome in colorectal cancer (CRC) (32). However, limited information is about the exact role of *GCNT2* and its methylation status in AML. In this study, we comprehensively analyzed the transcriptional expression of *GCNT2* and the DNA methylation profile in AML using multiple databases, including Gene Expression Omnibus (GEO), The Cancer Genome Atlas (TCGA) database, and web online resources in an effort to elucidate their clinical implications in AML.

## Materials and methods

### Assessment of *GCNT2* expression in normal hematopoiesis and AML cells

*GCNT2* expression profile in human normal hematopoiesis was analyzed using the gene-expression dataset (GSE42519) from GEO and a merged dataset HemaExplorer which consists of four datasets (GSE17054, GSE19599, GSE11864, and E-MEXP-1242) from BloodSpot (http://servers.binf.ku.dk/bloodspot/). For AML, we used three datasets (GSE13159, GSE24006, GSE30029), all of which contain AML samples and normal hematopoietic cell samples from bone marrow or umbilical cord blood, to compare the expression of *GCNT2* between AML and normal hematopoietic cell. Moreover, we downloaded normalized and combined RNAseq data of gene expression across pan-cancer (PANCAN, N=19131,G=60499) from the UCSC (http://xenabrowser.net), and extracted the data of *GCNT2* expression in AML samples (n=173) from TCGA database, and normal peripheral blood samples (n=337) from Genotype-Tissue Expression (GTEx) database.

### Prognostic analysis of *GCNT2* in AML

A high quality of survival data matched with each sample was acquired from the Pan-cancer Atlas paper (35), and follow-up data from the Therapeutically Applicable Research to Generate Effective Treatments (TARGET) database was supplemented. Tumors with less than 10 samples were removed, thereafter, a total of 44 tumor types that had matched *GCNT2* expression and overall survival data were subjected to Cox proportional hazards regression model. For AML, two independent AML cohorts [TCGA-LAML(n=202) and TARGET-AML(n=149)] with the follow-up data were aggregated to draw the survival curve by Kaplan‐Meier (KM) analysis.

### Assessment of *GCNT2* mutation in AML

The cBioPortal (http://cbioportal.org) is a web resource for cancer genomic analysis, and the multiple genomic data types from a mass of cancer studies were integrated by cBioPortal. We selected four studies that contained a bulk of AML samples to query the genomic alteration frequency of *GCNT2* in AML, these four studies are Pediatric Acute Myeloid Leukemia (TARGET, 2018; n=1025), Acute Myeloid Leukemia or myelodysplastic syndromes (WashU, 2016; n=136), Acute Myeloid Leukemia (OHSU, Nature 2018; n=672) and Acute Myeloid Leukemia (TCGA, Firehose Legacy; n=200).

### Analysis of *GCNT2* methylation in AML and normal hematopoiesis

For AML, we used three methylation datasets (GSE58477, GSE63409, and TCGA-LAML), in which, DNA methylation data (terms as beta value) was measured by Illumina Infinium HumanMethylation450 platform, and there were 34 CpG sites mapping onto the *GCNT2* gene. In GSE58477 and GSE63409, methylation profiles of AML samples and normal hematopoietic cells were available. Another study (GSE49618) has evaluated the methylation profile of normal healthy cells (monocytes, promyelocytes, polymorphonuclear cells, B cells, T cells, CD34+CD38- HSPCs). In our study, we extracted methylation data of *GCNT2* from the three independent studies.

In the TCGA-LAML cohort, RNAseq (IlluminaGA TCGA Hub, n=179)), DNA methylation (Methylation450k TCGA Hub, n=194), somatic mutation (wustl gene-level mutation call TCGA Hub, n=197), as well as corresponding clinical phenotypes (Phenotypes TCGA Hub, n=200), were downloaded from the UCSC, and the Tumor mutational burden (TMB), defined as the quantity of non-synonymous mutation in the genome, was downloaded via the cBioPortal.

The methylation-expression correlation curves were plotted by the SMART (http://www.bioinfo-zs.com/smartapp/), in which, the mRNA expression and DNA methylation data were derived from the TCGA project. We could also visualize the distribution of the 34-CpG and the *GCNT2* transcripts on the genomic structure by the SMART.

### Pathway and function enrichment analysis

To define the potential functions and underlying mechanism, the KEGG (Kyoto Encyclopedia of Genes and Genome) pathway enrichment and Gene ontology (GO) analyses were conducted via an online web tool DAVID (https://david.ncifcrf.gov/). The Gene Set Enrichment Analysis (GSEA) was applied to interpret the biological meanings associated with the difference of DNA methylation cohorts based on TCGA-AML RNAseq date. The Hallmark gene set, and the Reactome gene set derived from Molecular Signatures Database (http://www.gsea-msigdb.org/gsea/downloads.jsp) were included in the enrichment analysis. The gene set with the *P* value < 0.05 and FDR < 0.2 was deemed to be significantly enriched.

### Assessment of the immune microenvironment of AML

On basis of the gene expression profiles from a bulk of samples, various algorithms have been developed for accurate measurement of the tumor microenvironment.

In our study, the assessment of the tumor microenvironment was based on TCGA-AML RNAseq data. First, we employed the Estimation of Stromal and Immune cells in malignant tumors using the Expression data (ESTIMATE) (36) method to calculate the ImmuneScore, StromalScore, ESTIMATEScore of each AML sample, then, the exact immune cells abundance was assessed by five different algorithms, including CIBERCORT (37), EPIC (38), MCP-counter (39), quanTIseq (40), and xCell (41).

### Statistical analysis

The data was processed and statistically analyzed by using multiple platforms including Microsoft Excel 2019, IBM SPSS 22.0.0, GraphPad Prism 9.0, R-project 4.04, and an online service tool Sangerbox (http://www.sangerbox.com/tool). Data visualization was mainly carried out by Microsoft Excel 2019, GraphPad Prism 9.0, and Sangerbox. Differences in continuous variables between binary or multiple groups were analyzed using the Mann-Whitney U test or Kruskal-Wallis test, and categorical variables were compared by the chi-square test. The correlation coefficient was calculated whereby Person or Spearman analysis. For survival analysis, a univariate Cox proportional hazard regression model was conducted to evaluate the parameters that individually contribute to survival. The differences in OS between two stratified AML cohorts were analyzed by the Kaplan-Meier curve with the log-rank test. The Binary logistic regression analysis was used to analyze the independent factors that contribute to DNA methylation. A two-tailed *P*-value less than 0.05 was deemed statistical significance.

## Results

### Expression profile of *GCNT2* in human normal hematopoiesis and AML

**Figure 1:**
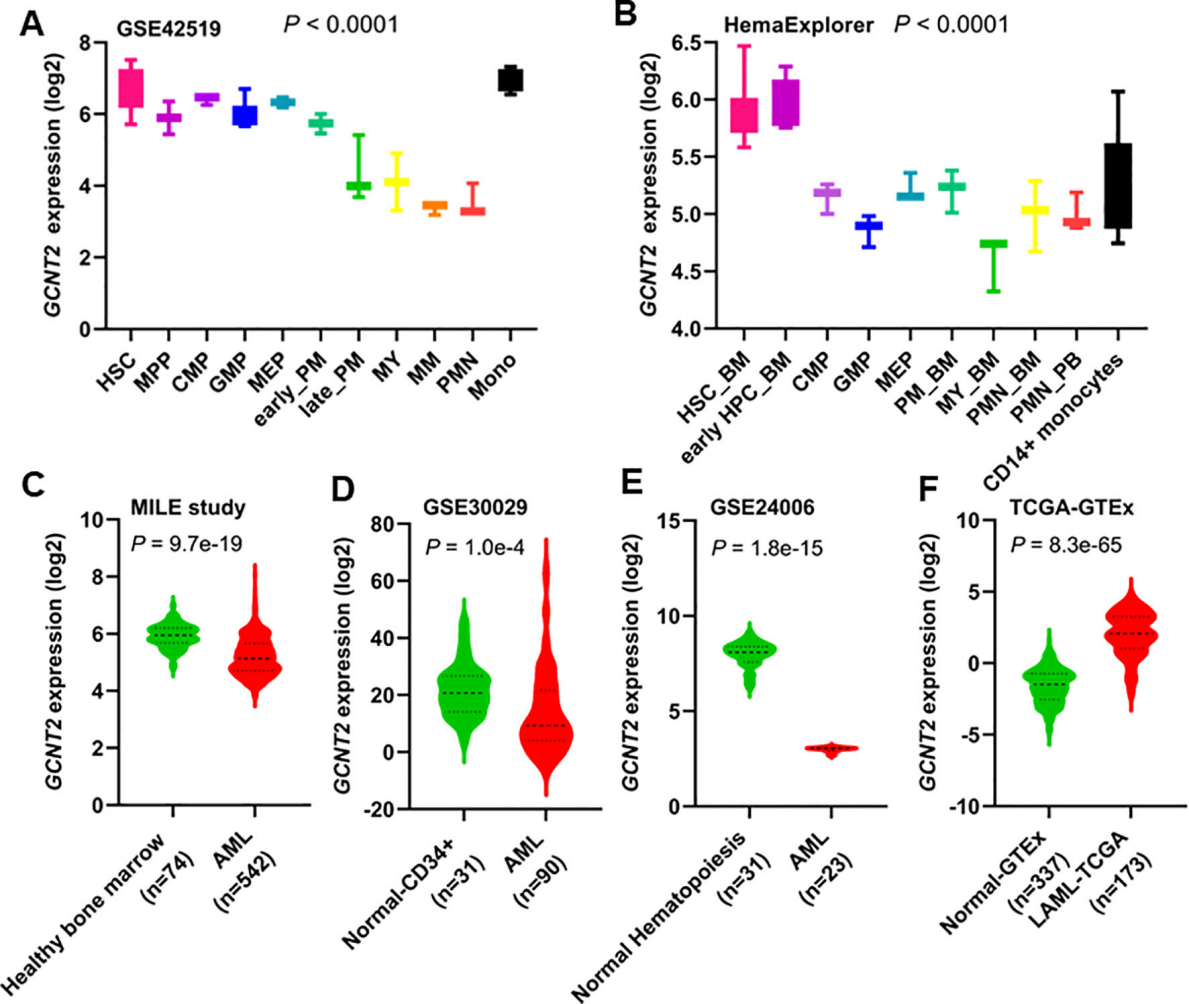
Expression of *GCNT2* in human normal hematopoiesis and AML. **(A)** The change of *GCNT2* during the hematopoiesis differentiation in GSE42519 data set (*P* < 0.0001, Kruskal-wallis test). **(B)** The change of *GCNT2* during the hematopoiesis differentiation in a merged data set HemaExplorer (*P* < 0.0001, Kruskal-wallis test). **(C–E)** Significantly reduced *GCNT2* was found in AML as compared to normal hematopoiesis from 3 independent studies **(C)** MILE study, **(D)** GSE30029, **(E)** GSE24006), **(F)** Remarkably increased *GCNT2* in AML (TCGA cohort) as compared to normal mature blood cells (normal-GTEx cohort). HSC, hematopoietic stem cell; MPP, multipotent progenitor; CMP, common myeloid progenitor; GMP, granulocyte−monocyte progenitor; MEP, megakaryocyte−erythrocyte progenitor; PM, promyelocyte; MY, myelocyte; MM, metamyelocytes; BC, band cell; PMN, polymorphonuclear cells; HPC, hematopoietic progenitor cells; BM, bone marrow; PB, peripheral blood.

The GSE42519 dataset involved the collection of bone marrow mononuclear cells from healthy donors, which were subsequently sorted into different stages of normal hematopoiesis using fluorescence-activated cell sorting (FACS). The expression pattern of *GCNT2* across the different stages of hematopoiesis was shown in (**Figure 1A**). We found that the *GCNT2* expression was highest in hematopoietic stem cells (HSC) and gradually decreased during the hematopoiesis differentiation; except for monocytes which retained a relatively high level of *GCNT2*, (*P <* 0.0001). A similar result was observed in a merged dataset (HemaExplorer), where, HSC and early hematopoietic progenitor cells (early HPC) exhibited a high expression of *GCNT2*, and the other committed progenitors and the more mature hematopoietic cell lineages showed a reduced expression of *GCNT2*; still, expression of *GCNT2* increased in the CD14+ monocytes (*P <* 0.0001), (**Figure 1B**). The dynamic change of *GCNT2* during the hematopoiesis differentiation suggests a potential role of *GCNT2* in HSC and monocytes, implying that *GCNT2* might be associated with stem cell-like feature and loss differentiated phenotype.

In AML, significantly reduced *GCNT2* was found in the three independent datasets (GSE13159, 542 AML vs. 74 normal, *P =* 9.7e-19; GSE30029, 90 AML vs. 31 normal, *P =* 1.0e-4; GSE24006, 23 AML vs. 31 normal, *P =* 1.8e-15), (**Figures 1C–E**). This unexpected result led us to hypothesize that *GCNT2* might still retain its function in immature hematopoietic cells. To further test this hypothesis, we compared *GCNT2* expression between AML cells and normally differentiated and mature peripheral blood cells. For this analysis, we utilized the TCGA-LAML cohort (n=173) and normal peripheral blood data from the GTEx project (n=337). Consistent with our hypothesis, we found that *GCNT2* expression was significantly higher in AML cells compared to differentiated and mature peripheral blood cells (*P =* 8.3e-65), (**Figure 1F**). These observations indicated that expression of *GCNT2* was repressed in AML as compared to normal premature hematopoietic cells, but remained substantially higher than mature blood cells.

### Expression of *GCNT2* positively correlates with stemness scores

**Figure 2:**
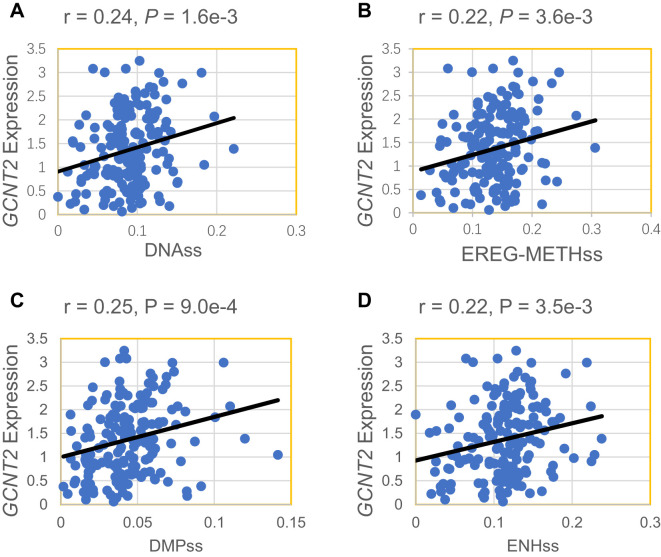
*GCNT2* expression positively correlated with 4 stemness indices. **(A)** DNAss DNA methylation-based (Stem cell signature probes (219 probes), that combines the 3 signatures). **(B)** EREG-METHss, Epigenetically regulated DNA methylation-based (87 probes). **(C)** DMPss Differentially methylated probes-based (62 probes). **(D)** ENHss Enhancer Elements/DNAmethylation-based (82 probes).

Our initial analysis of *GCNT2* expression revealed that it is highly expressed in early-stage hematopoietic cells, peaking in hematopoietic stem and progenitor cells. This observation suggests that the *GCNT2* gene may play a role in maintaining cellular stemness. To further substantiate this finding, we conducted a correlation analysis between *GCNT2* expression in the TCGA database and currently established stemness scores, DNA methylation-based Stemness Scores (DNAss) from a previous study (42), which has revealed that the higher stemness index, the more degrees of stemness. We correlated *GCNT2* expression with the four stemness indices (DNAss, EREG-METHss, DMPss, and ENHs), and notably, we found that the expression of *GCNT2* positively correlated with the four stemness indices (**Figure 2**). This result suggested that *GCNT2* played an important role in maintaining the stem cell-like property and oncogenic dedifferentiation in AML.

### Up-regulation of *GCNT2* predicts poor survival in AML

**Figure 3:**
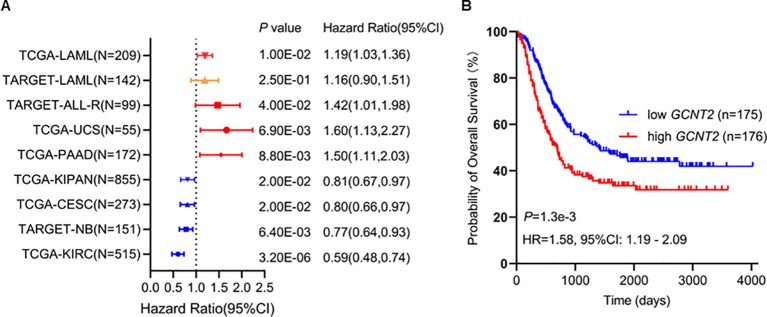
**(A)** *GCNT2* was identified to carry prognostic significance in 8 cancers by Cox proportional hazards regression model (*P* < 0.05), in TARGET-LAML cohort, the hazard ratio was 1.16, and the *P* value was 0.25. **(B)** Kaplan‐Meier (KM) analysis using the merged cohort (TCGA-LAML and TARGET-LAML) showed negative association of *GCNT2* with OS.

To explore the prognostic potential of *GCNT2* in AML, we first assessed the impact of *GCNT2* on overall survival (OS) from a pan-cancer study (35), notably, *GCNT2* was identified to carry prognostic significance in 8 cancers by Cox proportional hazards regression model (*P <* 0.05), those with Hazard Ratio (HR) >1 were TCGA-LAML (Acute Myeloid Leukemia), TARGET-ALL (Acute Lymphoblastic Leukemia), TCGA-UCS (Uterine Carcinosarcoma), TCGA- PAAD (Pancreatic adenocarcinoma), whereas, the HR<1 were observed in TCGA-KIPAN [Pan-kidney cohort (KICH+KIRC+KIRP)], TCGA-CESC (Cervical squamous cell carcinoma and endocervical adenocarcinoma), TARGET-NB (Neuroblastoma), TCGA-KIRC (Kidney renal clear cell carcinoma), **Figure 3A**. In the TARGET-LAML cohort, high-expressed *GCNT2* also conferred a worse outcome (*HR =* 1.16), although it had no statistical significance (*P =* 0.25), (**Figure 3A**). Then the two independent AML cohorts (TCGA-LAML and TARGET-LAML) containing a total of 351 samples were combined and subjected to Kaplan‐Meier analysis, it was revealed that patients with high *GCNT2* expression presented remarkably worse OS than those with low *GCNT2* expression (*P =* 1.3e-3, *HR =* 1.58), (**Figure 3B**).

In the TCGA-LAML cohort, the RNA sequencing data of 179 patients with detailed clinical information was downloaded from UCSC (IlluminaGA TCGA Hub). Two groups (*GCNT2* ^high^ and *GCNT2* ^LOW^) were stratified at the cutoff value of the median expression level, and the clinic characteristics of AML in between *GCNT2* ^high^ and *GCNT2* ^LOW^ groups were listed in **Table 1**. Statistical analysis revealed that *GCNT2* ^high^ group was characterized by lower hemoglobin concentration (*P =* 0.04), and a tendency of older age (*P =* 0.06). In FAB classification, the distributions of AML-FAB subtypes in *GCNT2* ^high^ and *GCNT2* ^LOW^ groups were obviously different (*P =* 0.000), where, FAB-M1-3 were more frequent in *GCNT2* ^low^ group, and FAB-M4-5 were more concentrated in *GCNT2* ^high^ group; as for cytogenetic abnormalities, patients with high *GCNT2* expression were significantly associated with complex karyotype (*P =* 0.0286), by contrast, low *GCNT2* expressed patients were more likely to carry some favorable abnormalities, such as t (15;17) (*P =* 0.0074) and t (8;21) (*P =* 0.0695); besides, the intermediate- and poor-cytogenetic risk groups contained a higher ratio of highly expressed *GCNT2*, as compared to the favorable-cytogenetic risk group (*P =* 0.0425). Concerning genetic abnormalities, a total of 176 patients with matched gene mutation information were available in the TCGA data set, and the top 10 mutation genes were displayed in **Table 1**. Notably, gene mutations of *RUNX1* and NRAS were more frequent in *GCNT2* ^high^ group (both of the p-values were 0.02), and the *CEBPA* gene mutation, regarded as a favorable biomarker in AML, tended to be more frequent in *GCNT2* ^LOW^ group (*P =* 0.09), however, the ratio of the patient with *FLT3* mutation more enriched in *GCNT2* ^LOW^ group, as unexpected.

**Table 1:** Clinicopathological feathers of AML patients with respect to the *GCNT2* expression.

Characteristics	Low *GCNT2* (n=89)	High GCNT2 (n=90)	*P*-value
Median age, year	55 (21-81)	60 (18-88)	***0.060***
Median hemoglobin, g/dL	10 (6-14)	9 (6-13)	**0.040**
Median leukocytes, ×10^9^/L	15 (0-297)	19 (1-137)	0.540
Median monocytes, ×10^9^/L	3 (0-76)	10.50 (0-76)	**0.000**
Median platelets, ×10^9^/L	42 (9-257)	52 (8-351)	0.230
Median BM_cellularity, %	90 (0-100)	90 (0-100)	0.520
Median BM_blast, %	45 (0-98)	17.50 (0-91)	**0.010**
Median BM_lymphocyte, %	19 (1-94)	18 (0-87)	0.470
Median BM_neutrophil, %	6 (0-46)	7 (0-94)	0.100
Gender, male/female	44/45	40/50	0.600
**FAB**			**0.000**
M0	6 (3.35%)	10 (5.59%)	0.3056
M1	27 (15.08%)	15 (8.38%)	**0.0309**
M2	27 (15.08%)	14 (7.82%)	**0.0186**
M3	14 (7.82%)	2 (1.12%)	**0.0009**
M4	8 (4.47%)	28 (15.64%)	**0.0002**
M5	5 (2.79%)	16 (8.94%)	**0.0115**
M6	0 (0.00 + 0%)	2 (1.12%)	0.1573
M7	1 (0.56%)	2 (1.12%)	0.5670
Not Classified	1 (0.56%)	1 (0.56%)	0.9937
**Cytogenetic abnormalities**			**0.020**
Normal	45 (27.95%)	44 (27.33%)	0.7797
t (15;17)	12 (7.45%)	2 (1.24%)	**0.0074**
t (8;21)	6 (3.73%)	1 (0.62%)	***0.0695***
inv (16)	3 (1.86%)	4 (2.48%)	0.6379
Trisomy 8	5 (3.11%)	3 (1.86%)	0.5251
Complex	7 (4.35%)	16 (9.94%)	**0.0286**
del (7q)/7q-	3 (1.86%)	4 (2.48%)	0.6379
del (7q)/7q-|t (9;11)	1 (0.62%)	0 (0%)	0.3308
del (5q)/5q-|del (7q)/7q-	0 (0%)	2 (1.24%)	0.1421
del (5q)/5q-	1 (0.62%)	0 (0%)	0.3308
t (9;11)	0 (0%)	2 (1.24%)	0.1421
N/A	6 (3.35%)	12 (6.70%)	
**Cytogenetics risk categories**			**0.210**
Favorable	21 (11.73%)	11 (6.15%)	**0.0425**
Intermediate/Normal	48 (26.82%)	54 (30.17%)	0.4574
Poor	17 (9.50%)	23 (12.85%)	0.3181
N/A	3 (1.68%)	2 (1.12%)	
**Gene mutations**			
* FLT3* mut, (+/-)	33/56	16/71	**0.009**
* NPM1* mut, (+/-)	23/66	25/62	0.790
* DNMT3A* mut, (+/-)	23/66	20/67	0.790
* IDH2* mut, (+/-)	10/79	7/80	0.640
* IDH1* mut, (+/-)	11/78	5/82	0.210
* RUNX1* mut, (+/-)	3/86	13/74	**0.020**
* TET2* mut, (+/-)	7/82	9/78	0.760
* TP53* mut, (+/-)	4/85	10/77	0.150
* CEBPA* mut, (+/-)	10/79	3/84	***0.090***
* NRAS* mut, (+/-)	2/87	11/76	**0.020**

FAB, French–American–British subtypes; BM, bone marrow; mut, mutation.

Bold values highlight statistically significant differences, bold italic values indicate a trend toward significance.

These results suggested an adverse prognosticator of *GCNT2* in AML, and the high frequency of over-expressed *GCNT2* in FAB-M4-5 was coincident with the result demonstrated above that monocyte had the unique expression profile of *GCNT2*.

### Genetic mutation of *GCNT2* in AML

**Figure 4:**
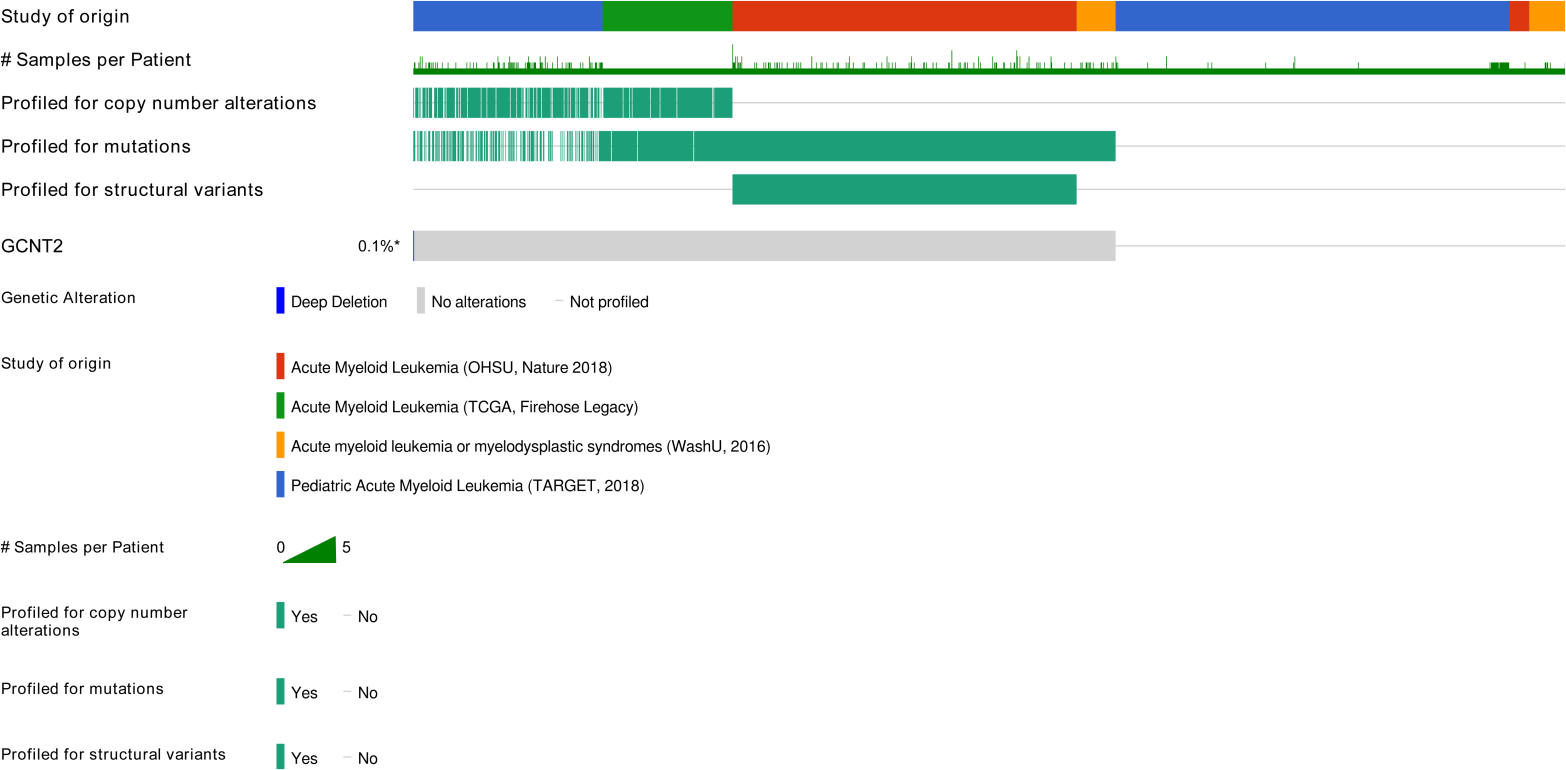
Genetic alteration of *GCNT2* was detected in 1 out of 1083 profiled samples (ratio: 0.1%) by querying 2033 AML samples in 4 studies via cBioPortal (http://cbioportal.org).

Since the loss function of *GCNT2* mutation was reported to critically link to many diseases, we quired the cBioPortal (http://cbioportal.org) to understand whether it was a player in AML. Four independent studies covering a total of 2033 AML samples were selected in cBioPortal, among which, genetic alteration of *GCNT2* was detected in 1 out of 1083 profiled samples (ratio: 0.1%) (**Figure 4**), therefore, we are able to conclude that regulation of *GCNT2* in AML may occur beyond *GCNT2* mutation.

### Methylation profile of *GCNT2* in AML

**Figure 5:**
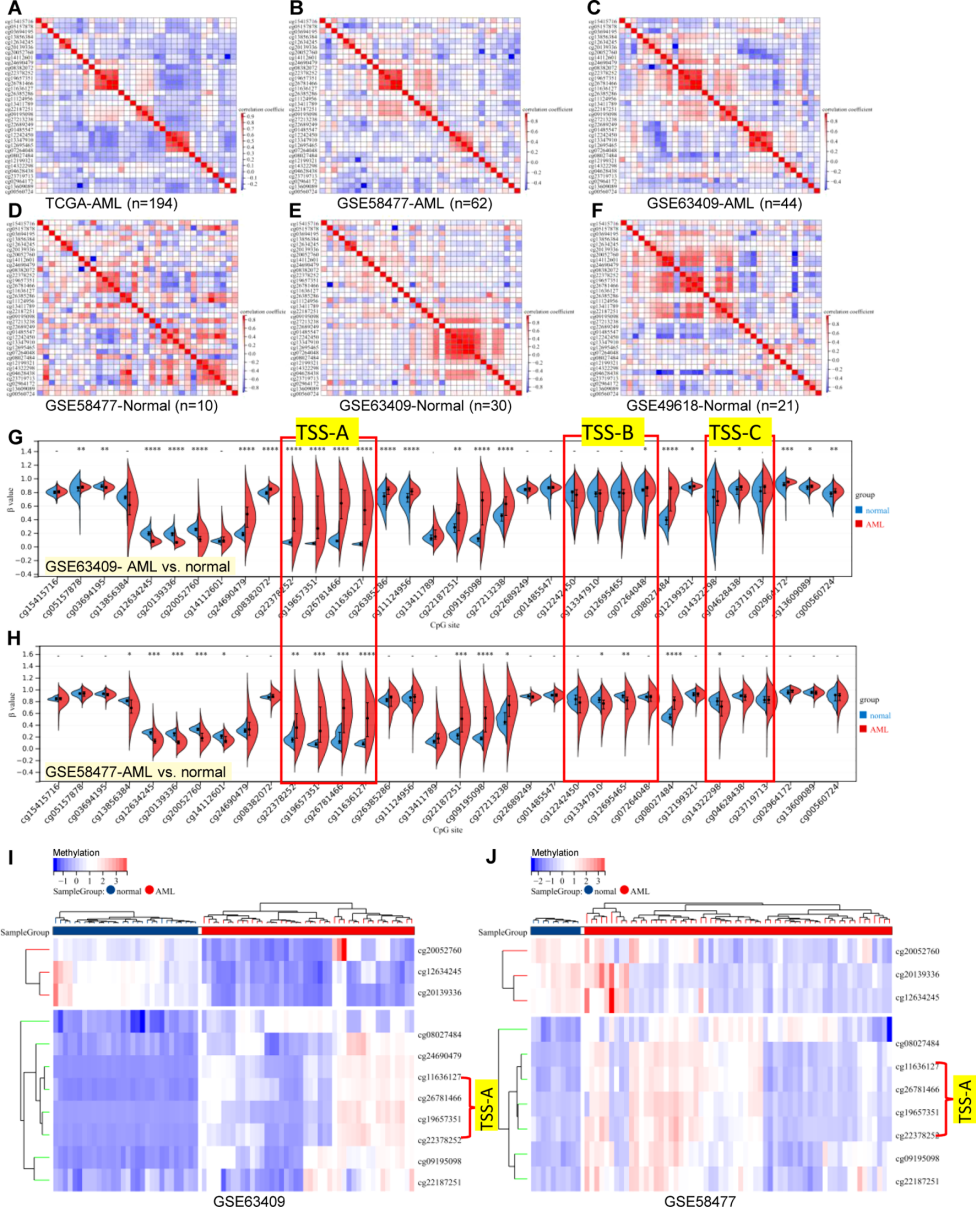
Correlation matrix of 34-CpG in AML and human normal hematopoietic cell. **(A–C)** three independent AML cohorts exhibited an accordant co-methylation manner [**(A)** TCGA-LAML, **(B)** GSE58477, **(C)** GSE63409], whereas, the methylation manners of normal hematopoietic cell in three independent studies were inconsistent [**(D)** GSE58477, **(E)** GSE63409, **(F)** GSE49618]. The global methylation profile of *GCNT2* by comparation analysis in between AML with normal controls, the distribution of abnormal methylation in AML was largely consistent in both datasets, both datasets exhibited abnormal methylation at TSS-A [**(G)** GSE63409, **(H)** GSE58477], The heatmap displays a consistent pattern of abnormally methylated loci across the samples [**(I)** GSE63409, **(J)** GSE58477]. *P < 0.05, **P < 0.01, ***P < 0.001, ****P < 0.0001.

Referring to the human genome version GRCh38/hg38, the *GCNT2* gene is located on chr6:10,492,223-10,629,368, spanning∼13.7 kb of genomic DNA. Previous studies have documented that there were 3 isoforms (isoforms A, B, and C), which were produced by alternative splicing, performing the main functions of *GCNT2* (20, 43, 44). They share the same exons 2 and 3, but with different exons 1 and promoter regions (43). To analyze the methylation status of GCNG2 in AML, the β value of the 34 CpG sites mapping onto the *GCNT2* gene were extracted from the three independent methylation studies (GSE58477, GSE63409, and TCGA-LAML). The annotation of the 34 probe IDs and their distances to transcriptional start sites (TSS) of the three isoforms were listed in **Table 2**. The correlation matrix from the TCGA-LAML cohort (n=194) disclosed that many adjacent CpG sites in different locations of the genomic DNA shared the parallel methylation status (co-methylation) toward each other. More importantly, most of the co-methylated CpG sites are near the transcription start sites (TSS) of the three isoforms. As shown in **Figure 5A**, the four sites (cg22378252, cg19657351, cg26781466, cg11636127) near the isoform A (TSS-A, -131∼132 distant to TSS) showed the highest co-methylation pattern, with the correlation coefficients (Pearson *r*) ranged 0.87-0.95; the second is the other four sites (cg12242450, cg13347910, cg12695465, cg07264048) near the isoform B (TSS-B, -86∼432, distant to TSS), with the correlation coefficients (Pearson *r*) ranged 0.83-0.93; the three CpG sites (cg14322298, cg04628438, cg23719713) near the isoform C (TSS-C, -188∼296, distant to TSS) showed a moderate co-methylation status (Pearson *r*: 0.36-0.64). This distinctive methylation manner was coincident with the other two independent AML cohorts (GSE58477, n=62; GSE63409, n=44), (**Figures 5B, C**). On the other hand, *GCNT2* methylation in the human normal hematopoietic cells from the three methylation studies (GSE58477, GSE63409, and GSE49618) showed inconsistent profiles (**Figures 5D–F**). In the comparison between AML and the normal control across the two independent datasets, GSE63409 and GSE58477, we examined the methylation levels of 34 CpG sites. Intriguingly, we observed a remarkable consistency in the distribution of abnormal methylation in AML as compared to normal cells across both datasets. Specifically, in certain distinct regions, notably the TSS-A region, both datasets demonstrated abnormal methylation at TSS-A (**Figures 5G–J**). Collectively, the CpG sites near the TSS of the 3 isoforms methylated with a synergistic process in AML, contribute to the unique epigenetic feature of AML.

**Table 2:** The annotation of the 34-CpG and their distances to transcriptional start sites (TSS) of the three isoforms of *GCNT2*.

Probe ID	CpG_beg	CpG_end	Dis-to-TSS (Isoform A)	Dis-to-TSS (Isoform B)	Dis-to-TSS (Isoform C)
cg15415716	10494553	10494554	26798	61454	91193
cg05157878	10494627	10494628	26724	61380	91119
cg03694195	10494766	10494767	26585	61241	90980
cg13856384	10494990	10494991	26361	61017	90756
cg12634245	10495131	10495132	26220	60876	90615
cg20139336	10495202	10495203	26149	60805	90544
cg20052760	10510556	10510557	10795	45451	75190
cg14112601	10520641	10520642	710	35366	65105
cg24690479	10520909	10520910	442	35098	64837
cg08382072	10521076	10521077	275	34931	64670
cg22378252	10521219	10521220	132	34788	64527
cg19657351	10521325	10521326	26	34682	64421
cg26781466	10521379	10521380	-28	34628	64367
cg11636127	10521482	10521483	-131	34525	64264
cg26385286	10527582	10527583	-6231	28425	58164
cg11124956	10528000	10528001	-6649	28007	57746
cg13411789	10528259	10528260	-6908	27748	57487
cg22187251	10529702	10529703	-8351	26305	56044
cg09195098	10529739	10529740	-8388	26268	56007
cg27213238	10529802	10529803	-8451	26205	55944
cg22689249	10555029	10555030	-33678	978	30717
cg01485547	10555031	10555032	-33680	976	30715
cg12242450	10555575	10555576	-34224	432	30171
cg13347910	10555620	10555621	-34269	387	30126
cg12695465	10555914	10555915	-34563	93	29832
cg07264048	10556093	10556094	-34742	-86	29653
cg08027484	10556290	10556291	-34939	-283	29456
cg12199321	10573237	10573238	-51886	-17230	12509
cg14322298	10585450	10585451	-64099	-29443	296
cg04628438	10585656	10585657	-64305	-29649	90
cg23719713	10585934	10585935	-64583	-29927	-188
cg02964172	10599226	10599227	-77875	-43219	-13480
cg13609089	10610458	10610459	-89107	-54451	-24712
cg00560724	10626737	10626738	-105386	-70730	-40991

### Transcriptional expression of *GCNT2* significantly correlated with DNA methylation

**Figure 6:**
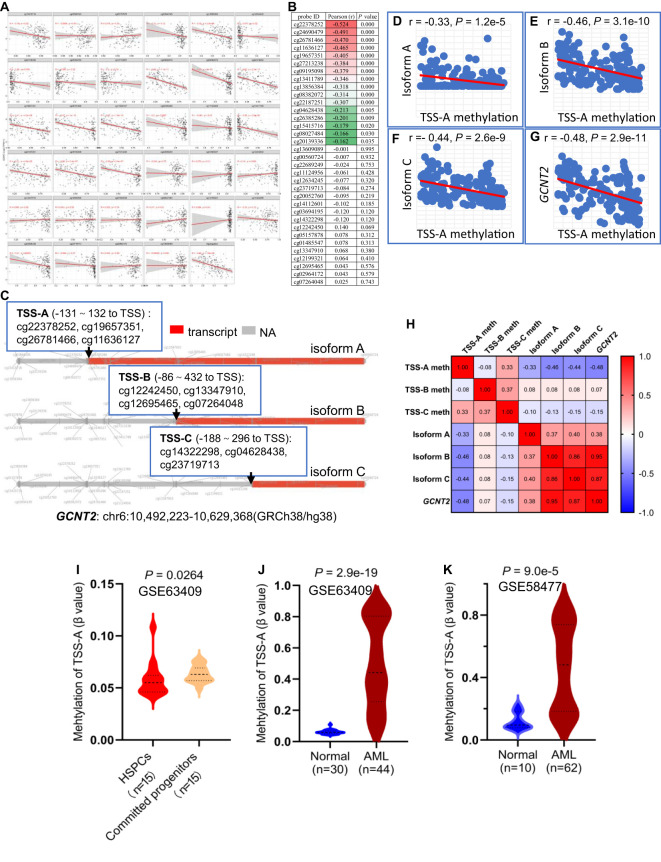
**(A)** Correlation analysis of *GCNT2* expression with each of the 34 CpG sites methylation via the SMART. **(B)** The ranked coefficients of the 34 CpG sites with *GCNT2* expression, the heatmap displayed the ranked CpG sites with *P* value < 0.05. **(C)** The genomic structure of the three isoforms **(A–C)** of *GCNT2* were plotted by the SMART, the methylation of TSS-A, TSS-B, and TSS-C was defined as the average of the co-methylated CpG sites near each of the TSS. **(D–G)** Correlation analysis of TSS-A methylation with transcriptional expression of the three isoforms **(A–C)**, and *GCNT2* in gene level. **(H)** The correlation heatmap indicated that the DNA methylation around the TSS of isoform A (TSS-A) significantly negatively correlated with all the three transcripts [isoform **(A–C)**], and *GCNT2* in gene level, expression of isoform B, isoform C and *GCNT2* in gene level exhibited a high level of co-expression. **(I)** Change of TSS-A methylation in HSPCs and committed progenitors in GSE63409 study. **(J, K)** Aberrant methylated TSS-A was indicated in AML from 2 studies [**(J)** GSE63409, **(K)** GSE58477].

To investigate the epigenetic modulation of *GCNT2* in AML, we correlated the *GCNT2* RNA-seq date with each of the 34 CpG sites methylations using the SMART, of note, 16 out of the 34 CpG sites showed significant negative correlation coefficients, still, the aggregation of the averaged methylation of the 34 CpG sites also demonstrated a remarkable negative correlation with *GCNT2* expression (*r =* -042, *P =* 1.8 e-08), (**Figure 6A**). The ranked coefficients of the 34 CpG sites were demonstrated in **Figure 6B**, and those with *P* value <0.05 were delineated in the heatmap. Interestingly enough, the top 5 CpG sites with a coefficient < -0.4 were comprised of the 4-CpG in TSS-A: cg22378252, cg19657351, cg26781466, cg11636127, the residue of cg24690479 with a coefficient of -0.491 is also close to the TSS of isoform A (442 distant to TSS, **Table 2**). The genomic structure of the three isoforms (A, B, C) was plotted by the SMART, and the approximate locations of TSS-A, TSS-B, and TSS-C were indicated in **Figure 6C**. Then, the methylation of TSS-A, TSS-B, and TSS-C was defined as the average of the co-methylated CpG sites near each of the TSS. Meanwhile, we also obtained the RNA-seq data of the three isoforms from the SMART for the correlation analysis. To our surprise, we found a significant inverse correlation between methylation of TSS-A with not only transcriptional expression of isoform A (Pearson r = -0.33, *P =* 1.2e-5, **Figure 6D**), but also with isoform B (Pearson r = -0.46, *P =* 3.1e-10, **Figure 6E**), isoform C (Pearson r = - 0.44, *P =* 2.6e-9, **Figure 6F**) and *GCNT2* in gene level (Pearson r = -0.48, *P =* 2.9e-11, **Figure 6G**), however, neither of TSS-B nor TSS-C methylation showed significant correlation with mRNA expression of each of the three isoforms and *GCNT2* (P > 0.05), (**Figure 6H**). Furthermore, expression of isoform B, isoform C and *GCNT2* in gene level presented strong positive correlation toward each other with the correlation coefficients (Pearson *r*) ranged 0.86-0.95, indicating a high level of co-expression, whereas mRNA expression of isoform A showed moderate correlations with isoform B (Pearson *r =* 0.37), isoform C (Pearson *r =* 0.40) and *GCNT2* (Pearson *r =* 0.38), (**Figure 6H**). This result revealed that DNA methylation around the TSS of isoform A (TSS-A) significantly negatively correlated with all three transcripts (isoform A, B, C) and *GCNT2* in gene level, and expression of isoform B and C predominantly represent the gene expression of *GCNT2*.

The aforementioned results showed repression of *GCNT2* in more mature hematopoiesis (compared with HSPC) and AML (compared with normal hematopoiesis), (**Figure 1**). To define whether the methylation of isoform A (TSS-A) is responsible for the effect, the differential methylation analysis was performed on basis of the two datasets: GSE63409, GSE58477. In GSE63409, a total of 30 normal hematopoiesis samples including 15 HSPCs (HSC, MPP, L-MPP) and 15 committed progenitors (CMP, GMP, MEP) were collected for the comparison analysis, and the result demonstrated TSS-A was hypomethylated in HSPCs (*P =* 0.00264), (**Figure 6I**); on the other hand, aberrant methylation of TSS-A was revealed in AML as compared to normal in two independent studies: GSE63409 (normal=30 vs. AML=44, *P =* 2.9e-19, **Figure 6J**), GSE58477 (normal=10 vs. AML=62, *P =* 9.0e-5, **Figure 6K**). These results suggested that methylation of TSS-A was responsible for the silence of *GCNT2* in mature hematopoiesis and AML.

### Hypomethylation of isoform a significantly predicts poor survival in AML

**Figure 7:**
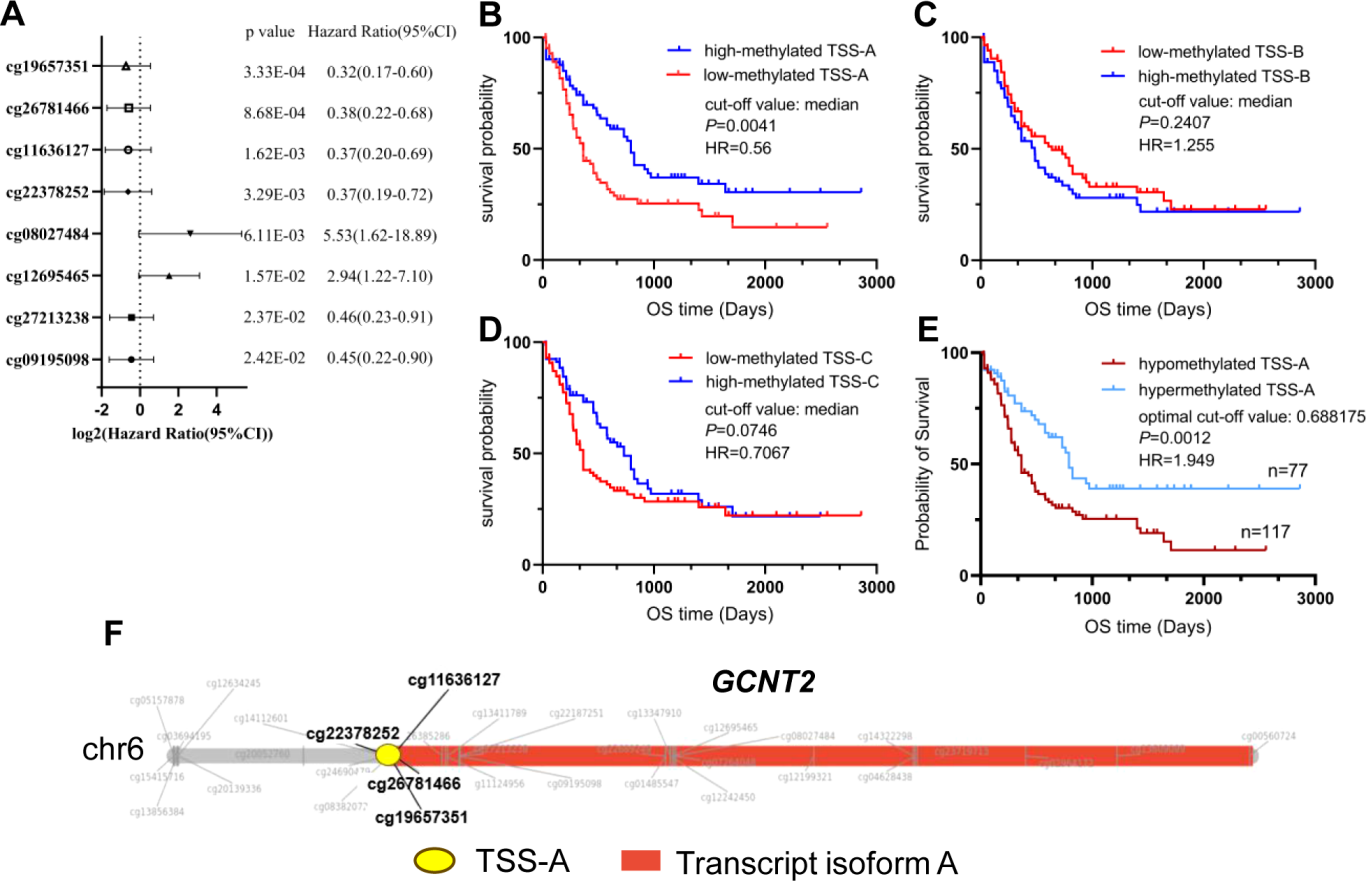
**(A)** Eight CpG sites having the prognostic potential were identified by the univariate Cox regression model. **(B–D)** Prognostic analysis of the three isoforms **(A–C)** methylation by the Kaplan-Meier Log-rank test. **(E)** Significantly different survival between TSS-A hypomethylation (n=117) and TSS-A hypermethylation (n=77) groups, which was divided using an optimal cut-off value of 0.688175. **(F)** The TSS-A region of the *GCNT2*, particularly focusing on four CpG sites (cg22378252, cg19657351, cg26781466, and cg11636127), is of high relevance to AML prognosis.

Hypomethylation of *GCNT2* leading to upregulation, that associated with tumor initiation, development, and metastasis, has been demonstrated in several malignancies (32–34). In CRC, the CpG island in the promoter of variant 2 (isoform B) is the key epigenetic regulator of *GCNT2*, and aberrant hypomethylation at this region contributed to a dismal outcome (32). To explore the value of methylation of *GCNT2* in contribution to prognosis in AML, the univariate Cox regression analysis was initially performed to assay each of the 34-CpG in contribution to overall survival from TCGA-AML cohort (n=194). Notably, a total of 8 CpG sites were identified to hold the prognostic potential (**Figure 7A**). More importantly, the top four CpG sites (cg19657351, cg26781466, cg11636127) which contributed to the positive prognosis (HR < 1) were all the members of TSS-A, implying that hypomethylation of TSS-A was associated with poor survival; the next two sites ((cg19657351, cg08027484) near the TSS-B indicated a negative prognosis; and the last two sites (cg27213238, cg09195098), also contributing to the positive prognosis (HR<1), belongs to TSS-C, **Figure 7A**. In Kaplan-Meier analysis with Log-rank test, high-methylated TSS-A conferred a significantly longer survival (*HR =* 0.5748, *P =* 0.0041, **Figure 7B**), however, neither TSS-B methylation (*P =* 0.2407, **Figure 7C**) nor TSS-C methylation (*P =* 0.0746, **Figure 7D**) showed any the prognostic impacts on overall survival. Then using the MaxStat method, an optimal cut-off value of 0.688175 was computed to divide patients into TSS-A hypomethylation (n=117) and TSS-A hypermethylation (n=77) groups, the comparison of the survival by K-M method between the two groups produced the HR of 1.949 (95%CI: 1.326 - 2.866, *P =* 0.0012, Log-rank test, **Figure 7E**). The methylation region specific to *GCNT2*, designated as TSS-A, which holds significance in relation to the prognosis of AML, is depicted in **Figure 7F**. The distributions of clinical characteristics between the two groups based on TCGA-AML cohort were displayed in **Table 3**. Statistical analysis disclosed that hypomethylated TSS-A was characterized by significantly elder age (*P =* 0.0002, higher platelet counts (*P =* 0.0001), lower level of BM cellularity (*P =* 0.02) and blast (*P =* 0.0017) but more infiltration of lymphocytes (*P =* 0.02); meanwhile, remarkable differences could be observed in the distribution of FAB classification between the two groups (*P =* 0.0065), hypermethylated TSS-A significantly correlated with FAB-M3 subtype (*P =* 0.007), whereas, hypomethylated TSS-A was significantly associated with FAB-M5 subtype (*P =* 0.0363); the cytogenetic abnormalities also exhibited a notably distinctive distribution (*P =* 0.000), in which, more frequency of complex karyotype was assembled in hypomethylation group (*P =* 0.003), however, the abnormalities of t (15;17) (*P =* 0.0301) and t (8;21) (*P =* 0.0014) were more frequent in hypermethylation group; furthermore, in cytogenetic risk stratification, hypermethylation of TSS-A was associated with favorable risk group (*P =* 0.0319) whereas hypomethylation of TSS-A correlated with poor risk group (*P =* 0.0059); in respect to genomic alterations, hypomethylated TSS-A was related to high TMB (*P =* 0.02), and for the top 10 mutated genes, patients with TSS-A hypomethylation were intimately associated with mutations of *DNMT3A* (*P =* 0.0008), *TP53* (*P =* 0.0018), *RUNX1* (*P =* 0.04), *WT1* (*P =* 0.05), by contrast, *CEBPA* gene mutation was determined to have more ratio in hypermethylation group (*P =* 0.0017). Then the Age, *DNMT3A*mu, *CEBPA*mu, *RUNX1*mu, *WT1*mu, inv (16), t (8;21), and t (15;17) were subjected to the Binary logistic regression analysis, we found that *DNMT3A*mu, *CEBPA*mu, *RUNX1*mu, *WT1*mu, inv (16), t (15;17) were independently associated with TSS-A methylation (*P* < 0.05, **Table 4**).

**Table 3:** Clinicopathological feathers of AML patients with respect to TSS-A methylation.

Characteristics	Hypomethylated TSS-A (n=117)	Hypermethylated TSS-A (n=77)	*P*-value
Median age, year	60 (21-88)	51 (18-76)	**0.0002**
Median hemoglobin, g/dL	9 (6-14)	9 (6-13)	0.4800
Median leukocytes, ×10^9^/L	15 (1-298)	28 (1-297)	0.3200
Median monocytes, ×10^9^/L	22 (1-94)	14 (0-91)	**0.0000**
Median platelet	61 (9-351)	36 (8-232)	**0.0001**
Median BM cellularity, %	90 (10-100)	90 (10-100)	**0.0017**
Median BM blast, %	17 (0-96)	48 (0-98)	**0.0200**
Median BM lymphocyte, %	22 (1-94)	14 (0-91)	**0.0200**
Median BM neutrophil, %	6 (0-94)	6 (0-46)	0.7700
Median TMB, Mut/Mb	0.33 (0-0.90)	0.27 (03-1.13)	**0.0200**
Gender (male/female)	67/50	38/39	0.3500
**FAB**			**0.0065**
M0	15 (7.73%)	4 (2.06%)	0.0897
M1	21 (10.82%)	21 (10.82%)	0.1229
M2	22 (11.34%)	20 (10.31%)	0.2354
M3	6 (3.09%)	13 (6.70%)	**0.0070**
M4	28 (14.43%)	14 (7.22%)	0.3414
M5	18 (9.28%)	4 (2.06%)	**0.0363**
M6	3 (1.55%)	0 (0%)	0.2782
M7	3 (1.55%)	0 (0%)	0.2782
Not Classified	1 (0.52%)	1 (0.52%)	1.0000
**Cytogenetic abnormalities**			**0.000**
Normal	58 (32.95%)	42 (23.86%)	0.5594
t (15;17)	4 (2.27%)	11 (6.25%)	**0.0061**
t (8;21)	0 (0%)	7 (3.98%)	**0.001**
inv (16)	8 (4.55%)	1 (0.57%)	***0.0684***
Trisomy 8	4 (2.27%)	5 (2.84%)	0.3319
Complex	21 (11.93%)	3 (1.70%)	**0.0002**
del (7q)/7q-	6 (3.09%)	1 (0.52%)	0.1549
del (7q)/7q-|t (9;11)	1 (0.52%)	0 (0%)	0.4118
del (5q)/5q-|del (7q)/7q-	2 (1.03%)	0 (0%)	0.2444
del (5q)/5q-	0 (0%)	1 (0.57%)	0.2204
t (9;11)	2 (1.14%)	0 (0%)	0.2444
N/A	12 (6.19%)	6 (3.09%)	
**Cytogenetic-risk categories**			**0.0075**
Favorable	16 (8.38%)	20 (10.47%)	**0.0319**
Intermediate/Normal	66 (34.55%)	47 (24.61%)	0.5402
Poor	33 (17.28%)	9 (4.71%)	**0.0059**
N/A	2 (1.03%)	1 (0.52%)	
**Gene mutations**			
* DNMT3A* mut (+/-)	40/77	9/68	**0.0008**
* CEBPA* mut (+/-)	2/115	11/66	**0.0017**
* TP53* mut (+/-)	16/101	0/77	**0.0018**
* RUNX1* mut (+/-)	14/103	2/75	**0.0400**
* WT1* mut (+/-)	3/114	8/69	**0.0500**
* NPM1* mut (+/-)	26/91	27/50	***0.0700***
* IDH1* mut (+/-)	10/107	9/68	0.6400
* NRAS* mut (+/-)	10/107	5/72	0.8000
* FLT3* mut (+/-)	32/85	23/54	0.8300
* TET2* mut (+/-)	9/108	7/70	0.9400

TSS-A, transcription start sites of GCNT2 isoform A; FAB, French–American–British subtypes; BM, bone marrow; TMB, tumor mutational burden; Mut/Mb, mutations per megabase.

Bold values highlight statistically significant differences, bold italic values indicate a trend toward significance.

**Table 4:** Binary logistic regression analysis of parameters associated with TSS-A hypomethylation.

Parameters	B	Sig.	Exp(B)	95% Confidence Interval	
				Lower Bound	Upper Bound
Age	0.028	**0.016**	1.028	1.005	1.052
DNMT3A mut	1.311	**0.004**	3.708	1.509	9.114
RUNX1 mut	1.949	**0.041**	7.018	1.082	45.514
CEBPA mut	-2.033	**0.018**	0.131	0.024	0.704
WT1 mut	-1.896	**0.021**	0.15	0.03	0.747
inv (16)	2.246	**0.048**	9.451	1.02	87.588
t (15;17)	-1.251	**0.049**	0.286	0.082	0.993
t (8;21)	-21.357	0.999	0	0	.

TSS-A, transcription start sites of *GCNT2* isoform A.

Bold values highlight statistically significant differences.

### DEGs and Enrichment analyses

**Figure 8:**
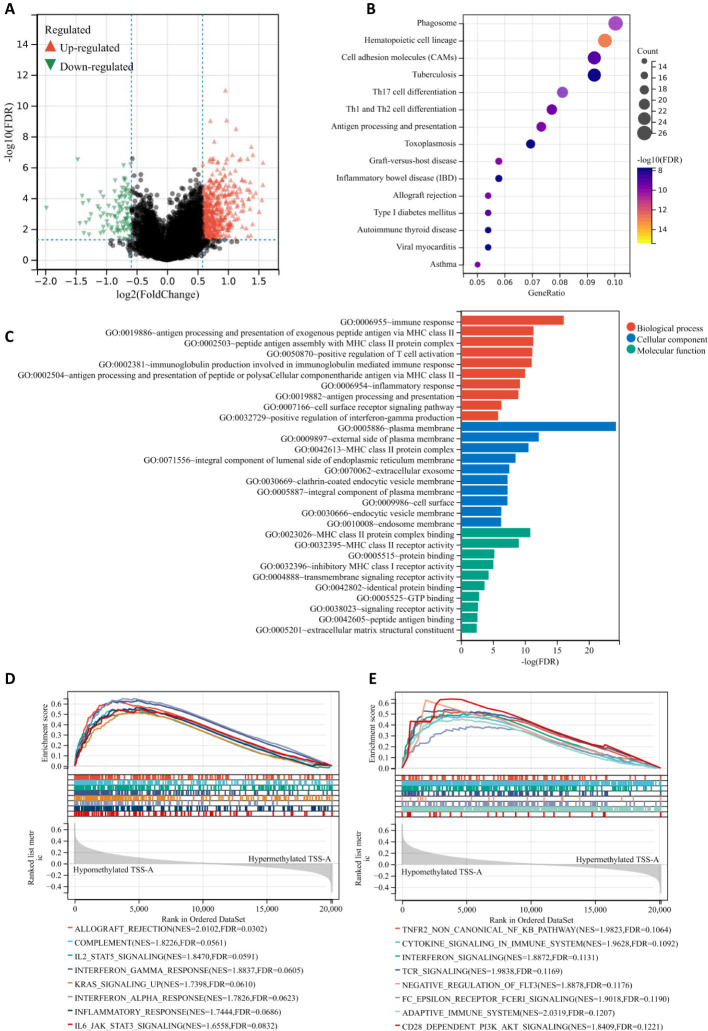
**(A)** Volcano plot of different gene-expression profiles between TSS-A hypomethylation and hypermethylation groups. **(B)** Bubble chart demonstrating the top 15 enriched KEGG pathways. **(C)** The bar graph demonstrating the top 10 enriched GO items (Biological process, Cellular component and Molecular function). **(D, E)** The GSEA results indicating the significantly enriched gene sets associated with TSS-A hypomethylation, **(D)** Analyzing with hallmark gene sets, **(E)** Analyzing with C2: Reactome gene sets.

In comparison between the TSS-A hypomethylation vs. hypermethylation groups using the limma test with the criteria of | FC | >1.5 and FDR < 0.05, we identified 553 differentially expressed genes (DEGs), including 464 up-regulated DEGs and 98 down-regulated DEGs (**Figure 8A**). Then KEGG pathway and GO function enrichment analyses of these DEGs were performed based on the DAVID (version 6.8), and a total of 33 KEGG pathways and 119 GO terms (including 59 BP items, 42 CC items, and 18 MF items) was obtained using a criterion of FDR < 0.05, (**Supplementary Table S1**). We drew the bubble chart to visualize the top 15 enriched KEGG pathways. As exhibited in **Figure 8B**, Hematopoietic cell lineage was the most enriched pathway, we could also discover many enriched pathways that related to immune response, such as Phagosome, Graft-versus-host disease, Antigen processing and presentation, Th1 and Th2 cell differentiation, Th17 cell differentiation, etc.

Moreover, the top 10 enriched terms (BP, CC, MF) were visualized in **Figure 8C**, Immune response (FDR *=* 8.52E-17), antigen processing and presentation of exogenous peptide antigen via MHC class II(FDR *=* 4.65E-12), peptide antigen assembly with MHC class II protein complex (FDR *=* 5.10E-12), positive regulation of T cell activation (FDR *=* 6.85E-12), immunoglobulin production involved in immunoglobulin mediated immune response (FDR *=* 8.60E-12), antigen processing and presentation of peptide or polysaccharide antigen via MHC class II (FDR *=* 9.08E-11), inflammatory response (FDR *=* 5.87E-10), antigen processing and presentation (FDR *=* 1.04E-09), cell surface receptor signaling pathway (FDR *=* 4.63E-07), positive regulation of interferon-gamma production (FDR *=* 1.51E-06) constituted the top 10 BP terms; for the CC, the DEGs mainly enriched in plasma membrane (FDR *=* 5.97E-25), external side of plasma membrane (FDR *=* 6.72E-13), MHC class II protein complex (FDR *=* 2.74E-11), integral component of lumenal side of endoplasmic reticulum membrane (FDR *=* 2.92E-09), extracellular exosome (FDR *=* 2.80E-08), clathrin-coated endocytic vesicle membrane (FDR *=* 5.10E-08), integral component of plasma membrane (FDR *=* 5.10E-08), cell surface (FDR *=* 5.33E-08), endocytic vesicle membrane FDR *=* 4.91E-07), endosome membrane (FDR *=* 5.27E-07); the top-ranked of MF terms included MHC class II protein complex binding (FDR *=* 1.456E-11), MHC class II receptor activity (FDR *=* 8.986E-10), protein binding (FDR *=* 6.00E-06), inhibitory MHC class I receptor activity (FDR *=* 8.72E-06), transmembrane signaling receptor activity (FDR *=* 4.67E-05), identical protein binding (FDR *=* 0.0002), GTP binding (FDR *=* 0.0014), signaling receptor activity (FDR *=* 0.0022), peptide antigen binding (FDR *=* 0.0027), extracellular matrix structural constituent (FDR *=* 0.0034). All these findings suggested that samples with hypomethylation of TSS-A were critically related to immune system, and that was also confirmed by the gene set enrichment analysis (GSEA), in which, for hallmark gene sets, hypomethylation of TSS-A was positively associated with gene sets prominently including Allograft rejection, Components, IL2/SATA5 signaling, IFN-gamma response, KRAS signaling, IFN-alpha response, Inflammatory response, and IL-6/JAK/STAT3 signaling (**Figure 8D**); for C2: Reactome gene sets, hypomethylated TSS-A enriched in a number of gene sets related to the innate, adaptive immune systems, and the cytokine signaling in immune system, such as TNFR2 non-canonical NF-kB pathway, Cytokine signaling, Interferon signaling, TCR signaling, Negative regulation of FLT3, Fc epsilon receptor (FCERI) signaling, Adaptive Immune System, and CD28 dependent PI3K/Akt signaling (**Figure 8E**). All these findings above suggested the pivotal role of the DNA methylation of *GCNT2* in immune regulation pathway in AML, and hypomethylation of TSS-A was involved in various processes of immune response, including antigen processing, presentation, recognition, and peptide antigen binding, cytokines/chemokines production, T cell activation, IFN-γ and INF-α signaling, etc.

### Immune infiltration profile of patients with TSS-A hypomethylation

**Figure 9:**
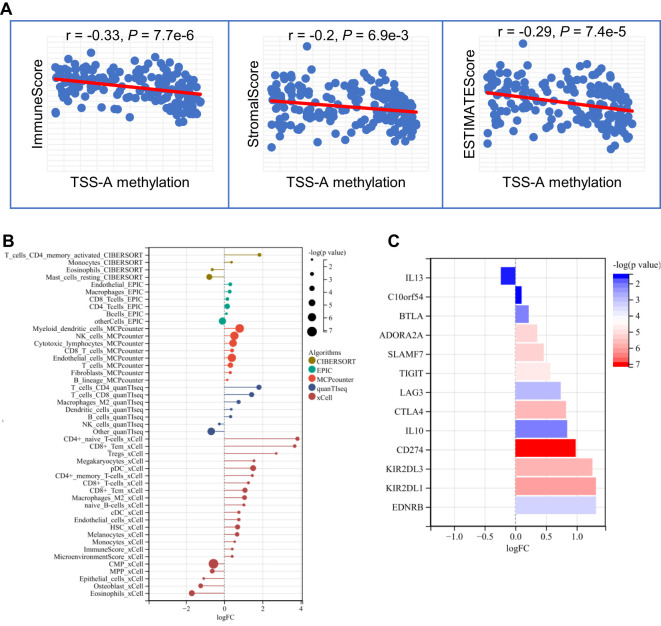
**(A)** TSS-A methylation inversely correlated with ImmuneScore, StromalScore, ESTIMATEScore computed by the ESTIMATE method. **(B)** Significant changes of various immune cells infiltrations by comparison of TSS-A hypomethylated vs. hypermethylated cohorts. The abundances of the immune cell were calculated using 5 different algorithms (CIBERCORT, EPIC, MCP-counter, quanTIseq, and xCell). **(C)** Significant changes of immune checkpoint molecules by comparing the TSS-A hypomethylated with hypermethylated cohorts, using the Mann-Whitney U test.

Since the important findings of hypomethylation of TSS-A in the immune system for AML from the enrichment analyses, it prompted us to explore the relationship between immune infiltration and TSS-A methylation. Multiple algorithms were implemented to evaluate the immune infiltration status of AML using TCGA RNA-seq data. First, using the ESTIMATE method (36), the ImmuneScore, StromalScore, ESTIMATEScore of each sample were calculated, and correlation analysis indicated that methylation of TSS-A was inversely correlated with ImmuneScore (co*r =* -0.33, *P =* 7.7e-6), StromalScore (co*r =* -0.2, *P =* 6.9e-3) and ESTIMATEScore (co*r =* -0.29, *P =* 7.4e-5), (**Figure 9A**). This result showed an intimate relationship between TSS-A methylation and immune infiltration, but the exact immune cells infiltration was not defined. Therefore, we next calculated the various immune cells infiltration of AML by employing 5 different algorithms, including CIBERCORT (37), EPIC (38), MCP-counter (39), quanTIseq (40), and xCell (41). By comparing the two AML cohorts (AML with TSS-A hypomethylation vs. AML with TSS-A hypermethylation) with t-tests, a distinctive immune infiltration was identified in TSS-A hypomethylated AML (**Figure 9B**): 1) much more types of immune cells were identified to enrich in TSS-A hypomethylated AML. In line with the ESTIMATE method, the higher ImmuneScore and MicroenvironmentScore were also indicated in xCell; 2) AML with TSS-A hypomethylation was significantly infiltrated with several anti-cancer immune cells, such as CD4+ T cells, CD8+ T cells, and dendritic cells, consistently in 2-4 different algorithms. The B cells, which administrate the humoral immunity, also showed higher infiltration in hypomethylated AML, which was validated in 4 different algorithms; 3) We could also observe hypomethylated AML presented more fraction of pro-cancer immune cells, macrophage M2 cells, and Tregs in quanTIseq and xCell, respectively; 4) the innate immunity participators (eosinophils, mast cells), by contrast, enriched in TSS-A hypermethylation, and the NK cells showed the opposite enrichments by MCPcounter and quanTIseq algorithms, respectively; 5) in accordance with the finding from the GEO and TCGA data above, information from xCell revealed that hypomethylation of TSS-A was associated with upregulation of HSC and monocytes, whereas hypermethylation of TSS-A was associated with the increment of CMP. These findings suggested that TSS-A hypomethylation was closely related to immune cells infiltration, both with activated immune cells (i.e. CD4+ T cells, CD8+ T cells, etc.) and immunosuppressive cells (i.e. Treg, macrophage M2 cells).

### Hypomethylation of TSS-A is associated with the upregulation of immune checkpoint inhibitors

It was believed that tumors could evade immunosurveillance via the expression of immune checkpoint molecules, antibody-based therapies that target the immune checkpoint molecules have archived great success in anti-tumor treatment (45). To explore the relationship between TSS-A hypomethylation and expression of the ICIs, a total of 24 inhibitory immune checkpoint genes, which were derived from former literature (46), were brought into our comparison analysis (TSS-A hypomethylation group vs. TSS-A hypermethylation group, using Mann-Whitney U test), notably, we found 13 out of the 24 ICIs differentially expressed (P < 0.05, **Figure 9C**), to our surprise, among the 13 differentially expressed ICIs, 12 ICIs were significantly upregulated. As displayed in **Figure 9C**, the top 5 significantly upregulated gene was *CD274* (PD-L1, *P =* 6.59E-08), followed by *KIR2DL1* (*P =* 6.93E-07), KIR2DL3 (*P =* 1.44E-06), *CTLA4* (*P =* 1.65E-06) and *SLAMF7* (*P =* 4.97E-06). This result revealed that AML with TSS-A hypomethylation might be impeded by the immune checkpoint inhibitors.

## Discussion

Recently, the posttranslational modulation of glycans on tumor cells has captured the attention of researchers. Aberrant glycosylation was considered as a novel hallmark of cancer, linking to multiple tumor-associated pathways such as proliferation (25), migration (23), immune evasion 13], and distant metastasis (13, 14). An emerging glycosyltransferase *GCNT2*, which catalyzes I-branched N-glycans, was documented to be associated with various cancers, acting as an oncogene or a tumor suppressor in different cancer types. A recent study has demonstrated a protective effect of *GCNT2*/I-branched glycans in melanomas, with experiments *in vitro* and *in vivo* demonstrating that *GCNT2*/I-branched glycans promoted melanoma growth and survival through IGF-1 and ECM-mediated signaling pathways (25). In fact, the oncogenic function of *GCNT2* was reported in the most investigated cancers, including breast cancer, prostate cancer, and esophageal carcinoma (23, 24, 26). An intensive study on breast cancer has addressed the underlying mechanism of the TGF-β signaling pathway and epithelial-mesenchymal transition (EMT) induced by *GCNT2* (26). On the other hand, aberrant methylation of *GCNT2* was reported to be responsible for the silence of *GCNT2* in CRC, and hypomethylation of *GCNT2* isoform B was closely associated with disease progress and metastasis (32).

In the present study, we focused on *GCNT2* and its epigenetic regulation in AML. In human normal hematopoiesis cells, we observed that expression of *GCNT2* gradually decreased during the HSCs differentiation to terminal effector cells, with exception of monocytes, suggesting that *GCNT2* might carry the potential in maintaining the multipotency, dedifferentiation, and self-renewal of HSC while its down-regulation induced HSCs differentiating toward to their progeny; the retaining *GCNT2* in monocytes might harbor some special function worthy of further investigation. In the context of AML pathogenesis, the high expression of *GCNT2* in hematopoietic stem and progenitor cells implies a potential role in the self-renewal and proliferative capacity of leukemic stem cells (LSCs). To further substantiate this finding, we correlated GCNT2 expression with currently established stemness scores in the TCGA database. Our analysis revealed a positive correlation between GCNT2 expression and stemness scores, thus providing additional evidence to support the role of *GCNT2* in maintaining cellular stemness. It is well-established that LSCs are a major obstacle in the successful treatment of AML, as they are resistant to conventional therapies and capable of reinitiating the leukemic process. Therefore, the identification of *GCNT2* as a potential regulator of stemness in hematopoietic cells raises the possibility that it could be a therapeutic target for disrupting the self-renewal and survival mechanisms of LSCs.

In AML, expression of *GCNT2* was remarkably decreased in comparison to normal bone marrow, yet remained much higher compared to mature blood cells. The survival analysis on basis of two AML cohorts disclosed that upregulation of *GCNT2* conferred an adverse outcome in AML; data from the TCGA cohort indicated that high-expressed *GCNT2* correlated with multiple adverse phenotypes, such as lower HB, older age, complex karyotype, *RUNX1* and NRAS mutations, and patients with *GCNT2* ^low^ tended to have more incidence of *CEBPA* mutation regarding as a favorable biomarker; besides, patients with FAB-M_4,5_ were more frequent with high *GCNT2* expression in accordance with the observation above that expression of *GCNT2* was reserved in the monocytes. These results suggested the oncogenic driver of *GCNT2* in AML, and the down-regulation of *GCNT2* in AML could be explained by the fact that the silence of *GCNT2* was a protective effect, whereas the reactivation of *GCNT2* indeed contributed to a worse outcome.

It has been reported that genetic mutation of *GCNT2*, leading to loss of *GCNT2* expression, was responsible for the disease of adult i phenotype and congenital cataracts (19–21). In AML however, data from cBioPortal indicated that the incidence of *GCNT2* mutation was only 0.1%. On the other hand, studies either on normal erythroid hematopoiesis (31) or on many solid tumors have shown a critical regulatory mechanism of *GCNT2* on an epigenetic level, regarding DNA methylation. In the tumor, aberrant *GCNT2* methylation was closely associated with tumor development by controlling *GCNT2* expression (32–34). Indeed, our findings from the GEO database confirmed that DNA methylation was conclusively responsible for the silence of *GCNT2* both in HSC differentiation and AML. This result gained a new insight that the putative stem cell-like function of *GCNT2*, acting as an adverse factor in AML, was suppressed by DNA methylation, and its reactivation by hypomethylation conferred the worse phenotype. A similar situation has been observed in CRC, where reduced *GCNT2* was found in primary tumor tissues compared to corresponding normal mucosa, however, DNA hypomethylation inducing *GCNT2* expression enhanced progression and led to poor survival (32).

Correlation analysis on the TCGA AML cohort has shown a substantially negative correlation of DNA methylation with *GCNT2* transcriptional expression. Interestingly, we found that 11 CpG sites, that distributed in the three different regions (TSS-A, TSS-B, TSS-C), exhibited a co-methylation pattern, and the loci with paralleled methylation of CpG sites are neighboring and principally located in three distinct regions around the TSS of the three isoforms (A, B, and C). The way of co-methylation process influenced by structural factor has been evidenced and observed by emerging studies (47, 48). This distinct co-methylation pattern found in the TCGA-AML cohort was consistent with the other two independent AML cohorts (GSE58477, GSE63409), totally differing from that in the normal cohort, and shedding the light on the unique methylation profile of AML.

It has been documented that the determinant of transcriptional expression on the epigenetic level was the DNA methylation of the first exon (49). With regard to *GCNT2*, there are three different 1^st^ exons belonging to isoforms (A, B, C). Nakamura K, et al. (32) have shown that methylation of variant 2 (isoform B) was critical for *GCNT2* expression, and demethylation of variant 2 by 5 Aza-dC treatment could induce expression of all the three variants (or isoform A, B, and C) in CRC cell lines (32); hypomethylation of *GCNT2* variant 2 was deemed to be the aggressive phenotype, significantly predicting dismal prognosis (32). Herein, we have demonstrated that the transcripts of isoform B and C predominantly represented the expression of *GCNT2* (*r =* 0.95 and *r =* 0.87, respectively) in AML, however, it was methylation of isoform A, rather than isoform B, substantially correlated with the transcriptional level of isoform A, B, and C (*r =* -0.46, *P =* 3.1e-10 and *r =* -0.44, *P =* 2.6e-9, respectively), as well as the *GCNT2* in gene level (*r =* -0.48, *P =* 2.9e-11). Therefore, the co-methylation CpG sites around the TSS of isoform A was the definitive factor driving the transcriptional expression of *GCNT2*. These findings suggest that, while there may be some overlap in their regulation, isoform A may also be subject to distinct regulatory influences that differentiate it from the other isoforms, and further study is needed to address the detailed mechanisms underlying these observations. Understanding the precise molecular pathways and interactions that lead to the altered methylation patterns of *GCNT2* variants in AML could provide crucial insights into the progression and prognosis of this disease. Such research may involve in-depth epigenetic analysis, including the investigation of histone modifications and chromatin structure, as well as the identification of potential regulatory factors and signaling pathways involved in modulating *GCNT2* methylation. Additionally, functional studies to assess the impact of these methylation changes on *GCNT2* expression and function are essential to fully elucidate the biological significance of these findings. Likewise in prognosis concerning, we have dissected that hypomethylation of isoform A significantly predicted a poor outcome in AML. This was quite different from CRC, in which hypomethylation of isoform B was the poor prognosticator (32). In clinical implication, hypomethylation of isoform A was associated with the higher level of platelet and bone marrow lymphocyte and TMB, but the lower level of bone marrow blast and cellularity and more tendency of inv (16) and del (7q)/7q-, both *P =* 0.086; by contrast, hypermethylation of isoform A was related to more frequency of t (8:21) (*P =* 0.0014) and t (15;17) (*P =* 0.0301). In AML, the two unique subtypes: FAB-M2 with t (8:21) generating *RUNX1*-*RUNX1*T1 fusion gene, and APL or M3 with t (15;17), generating PML-RARA fusion gene, were demonstrated to interact with *DNMT3A*, leading to aberrant DNA methylation that contributed to leukemogenesis (50–52). Therefore, we suggested that both of the two fusion genes targeted *GCNT2* by affecting *DNMT3A*, resulting in *GCNT2* hyper-methylation. It’s also worth mentioning that 8 out of 9 patients bearing inv (16) belonged to the hypomethylated cohort (*P =* 0.08). AML with inv (16) is a distinctive subtype with a morphology of increasing myelomonocytes and immature basophilic granules, as was classified into FAB-M4eo (53). The *CBFB-MYH11* fusion generated by inv (16) was documented to inhibit *DNMT3A* by disrupting recruiting *RUNX1* to bind target genes, thus contributing to hypomethylation (54). In agreement with this finding, our concerned AML cohort with inv (16) conferred hypomethylation of *GCNT2* and consequently led to upregulation of *GCNT2*, which was also supposed to be the molecular hallmark of monocytes in normal hematopoiesis as mentioned above.

Moreover, a hand of hotspot somatic mutations in AML were identified to notably correlate with methylation of *GCNT2* isoform A (*DMNT3A*, *CEBPA*, *RUNX1*, *WT1*, *TP53*). Among these, *DNMT3A*, *CEBPA*, *RUNX1*, and *WT1* have been well recognized to interplay and involve in DNA methylation that resulted in AML initiation and development (54–57). *DNMT3A* is an important gene encoding a DNA methyltransferase, which is responsible for DNA methylation in mammalian development (58). In AML, *DNMT3A* mutation was identified to be one of the most frequent somatic mutations, significantly conferring a worse survival (59). Function experiment has verified the mutation of *DNMT3A* in disrupting the ability of the methyltransferase (60). In line, *DNMT3A* mutation-associated *GCNT2* demethylation was observed in our study, let alone mutation leading to dysfunction of *DNMT3A*, we also supposed that mutation-related suppression of *DNMT3A* may be another reason. As a matter of fact, based on the TCGA-LAML, a notable demethylation of TSS-A (*P* = 3.0e-5) and downregulation of *DNMT3A (P* = 1.2e-3) was observed in DNMT3A-mut group (**Supplementary Figure S1A**), furthermore, expression of *DNMT3A* was positively correlated with TSS-A methylation (r = 0.25, *P* = 9.4e-4, **Supplementary Figure S1B**), and significantly negatively correlated with *GCNT2* expression (r= -0.32, *P* = 1.1e-5, **Supplementary Figure S1C**). In AML, *CEBPA* mutation is related to cytogenetically normal AML (CN-AML), mutation in *CEBPA* alone predicts a positive prognosis. A recent study had evidenced that *CEBPA* could inhibit *DNMT3A* activity, mutation in *CEBPA* resulted in the loss of the inhibitory capacity thereby leading to aberrant gene methylation (57). On the other hand, studies also revealed that the transcript factor of *CEBPA* could guide *TET2* demethylase to the binding site, causing DNA demethylation, and mutation in *CEBPA* hence causing DNA hypermethylation (56). *RUNX1* is another transcript factor gene that interacts with both methyltransferase (*DNMT3A*) (54) and demethylase (TET) (61) bringing about methylation or demethylation. Although *RUNX1* mutation was reported to promote hypermethylation in AML (56), however, we observed that *RUNX1* mutation was associated with *GCNT2* hypomethylation, assuming that guiding *DNMT3A* to the binding site by *RUNX1* and leading to *GCNT2* methylation was blocked by the mutation in *RUNX1*. Another piece of evidence is that the *RUNX1*-*RUNX1T1* fusion protein stemming from t (8;21) retained the binding site of *RUNX1* which promoted methylation of target genes in AML (50, 62). Likewise, as a transcript factor, *WT1* recruits *TET2* to epigenetically activate target genes by demethylation (63), and mutant *WT1* was evidenced to produce DNA hypermethylation in the AML cell line, typically targeting polycomb repressor complex 2 (PRC2) (55). This phenomenon was also pronounced for *GCNT2* methylation in AML, where patients with *WT1* mutation were enriched in hypermethylation of *GCNT2* isoform A. In our Binary logistic regression model, age, inv (16), t (15;17), genes mutations of *DNMT3A*, *CEBPA*, *RUNX1*, and *WT1* were deemed to independently correlate with methylation of *GCNT2* isoform A, suggesting that the diverse phenotypic features of AML, which were attributed by distinctive cytogenetic or molecular abnormalities, came in the way of regulating *GCNT2* methylation. Our research highlights the central role of *GCNT2* methylation in the pathogenesis of AML.

Besides, we have demonstrated that AML with hypomethylated isoform A (TSS-A) presented a lower level of bone marrow blast and cellularity, but more infiltration of bone marrow lymphocytes, and carried higher TMB. So, we supposed that CpG methylation near the TSS of isoform A played an important role in the immune microenvironment of AML. In order to define the potential functions and underlying mechanisms that correlated with TSS-A methylation, we performed GO, KEGG enrichment analyses, and GSEA. Indeed, we found that TSS-A hypomethylation was closely involved in various processes of the immune system, activating multiple immune regulation pathways, such as IL2/SATA5 signaling, IFN-gamma and -alpha response, KRAS signaling, CD28 dependent PI3K/AKT signaling, T cell activation, Cytokine signaling, etc. Furthermore, the immune infiltration analyses on basis of RNA sequencing data have also revealed that AML with hypomethylation of TSS-A linked to higher immune cells infiltration, predominately with adaptive immune cells (e.g. CD4+ T cells, CD8+ T cells, and dendritic cells, etc.). Meanwhile, TSS-A hypomethylated AML was also observed to be associated with more abundance of Treg and macrophage M2 cells, which were deemed to mediate immune tolerance and evasion, acting as suppressors of anti-tumor immunity (64, 65). This immune infiltration pattern was in accord with the T-cell-inflamed signature, which was defined on the basis 18-gene expression profile (66). In T-cell-inflamed tumor microenvironment, the function of effector T cells was inhibited by many factors, such as Tregs or immune checkpoint molecules (e.g. PD-L1, IDO, etc.). As a matter of fact, we have found that multiple inhibitory immune checkpoint molecules were increased in TSS-A hypomethylated cohort, including CD274 (PD-L1), and CTLA4. Ongoing studies have explored the monoclonal antibody (mAb)-based immune checkpoint blockade (ICB) treatment for AML by targeting CTLA4 or PD1/PD-L1 pathway (67). However, identifying the patients who will respond to the immunotherapy remains a challenge. Cancer with high TMB will produce more neo-antigens, resulting in greater chances to trigger T-cell immune response, and TMB has been developed as a biomarker for predicting immunotherapy response (68). The current studies have confirmed that patients with T-cell-inflamed phenotype, high TMB, and expression of PD-L1 were more likely to benefit from immunotherapy (66, 69). Here, our study uncovered that TSS-A hypomethylated AML was characterized by higher TMB, more infiltration of immune cells, and upregulation of immune checkpoint genes, thus, we are able to suggest hypomethylation of TSS-A could be served as a useful biomarker to identify AML patients who will respond to immunotherapy.

Overall, we have illustrated the pro-stemness role of *GCNT2* which was associated with adverse outcomes in AML and methylation of *GCNT2* exhibits a distinctive manner that the CpG sites around the TSS of the three isoforms were co-methylated. In AML, the silence of *GCNT2* was attributed to DNA methylation of isoform A (TSS-A). Hypomethylation of TSS-A significantly predicted poor survival, linking to multiple cytogenetic and molecular abnormalities, which were well documented to be implicated in the regulation of DNA methylation. Furthermore, methylation of TSS-A intimately correlates with the immune system, and AML with hypomethylated TSS-A presents a higher immune cell infiltration, having the T-cell inflamed phenotype, and is more likely to benefit from immunotherapy. However, this study had several limitations. Firstly, as a retrospective study based on public databases, our findings on *GCNT2* expression and methylation as potential biomarkers for AML prognosis and clinical guidance requires further validation with more extensive sample sizes and prospective studies. Secondly, additional experimental and clinical studies is needed to ascertain whether targeting these mechanisms can enhance therapeutic outcomes. Lastly, the data pertaining to immune infiltration profiles linked with TSS-A hypomethylation status warrants further exploration to determine their practical utility in personalized treatment strategies. More thorough studies are required to tackle these concerns in the future. Our study has shed new insight into the relationships of genetic, epigenetic alterations, and glycosylation modification involved in AML.

## Data Availability

The datasets presented in this study can be found in online repositories. The names of the repository/repositories and accession number(s) can be found in the article/**Supplementary Material**.
