# Supplementary figures and images for: Hypomethylation of GCNT2 isoform A correlates with transcriptional expression and is associated with poor survival in acute myeloid leukemia
Source: Front Immunol. 2025 Feb 17;16:1490330. doi: 10.3389/fimmu.2025.1490330 (PMC11873079; doi:10.3389/fimmu.2025.1490330)

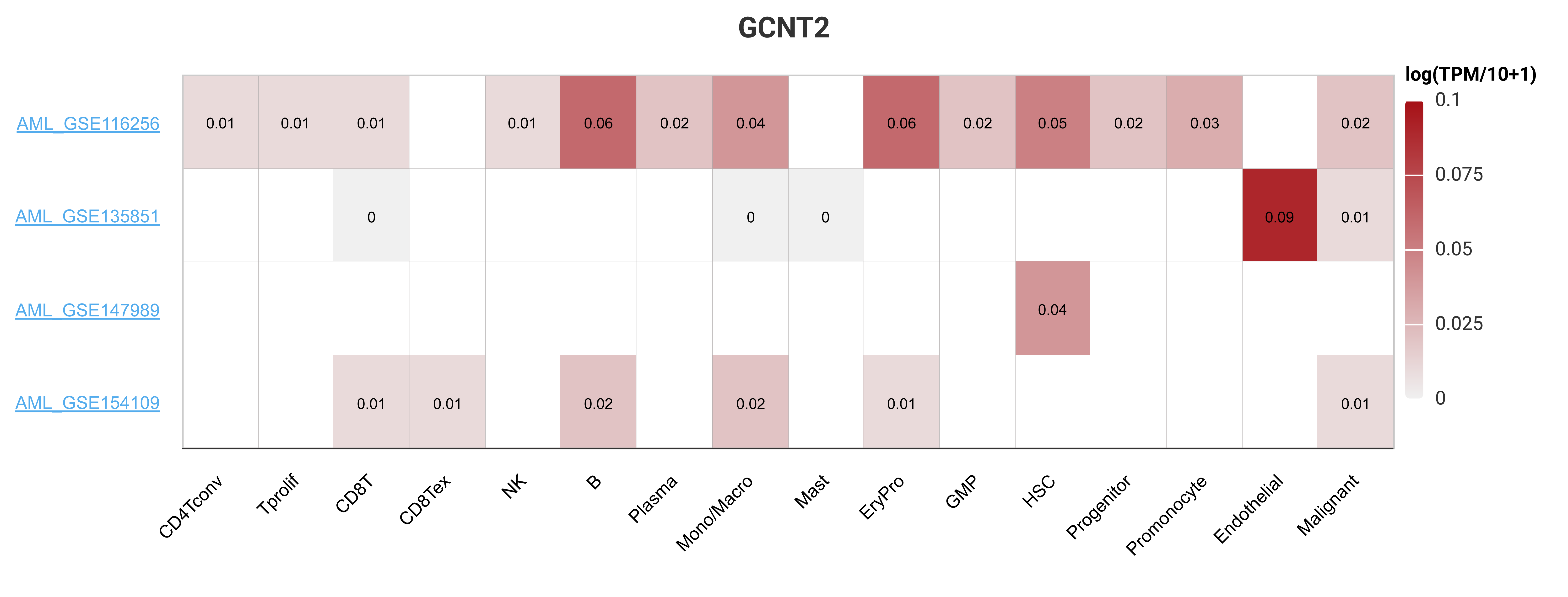

Supplement: Supplementary file 1 [file Image1.tif]

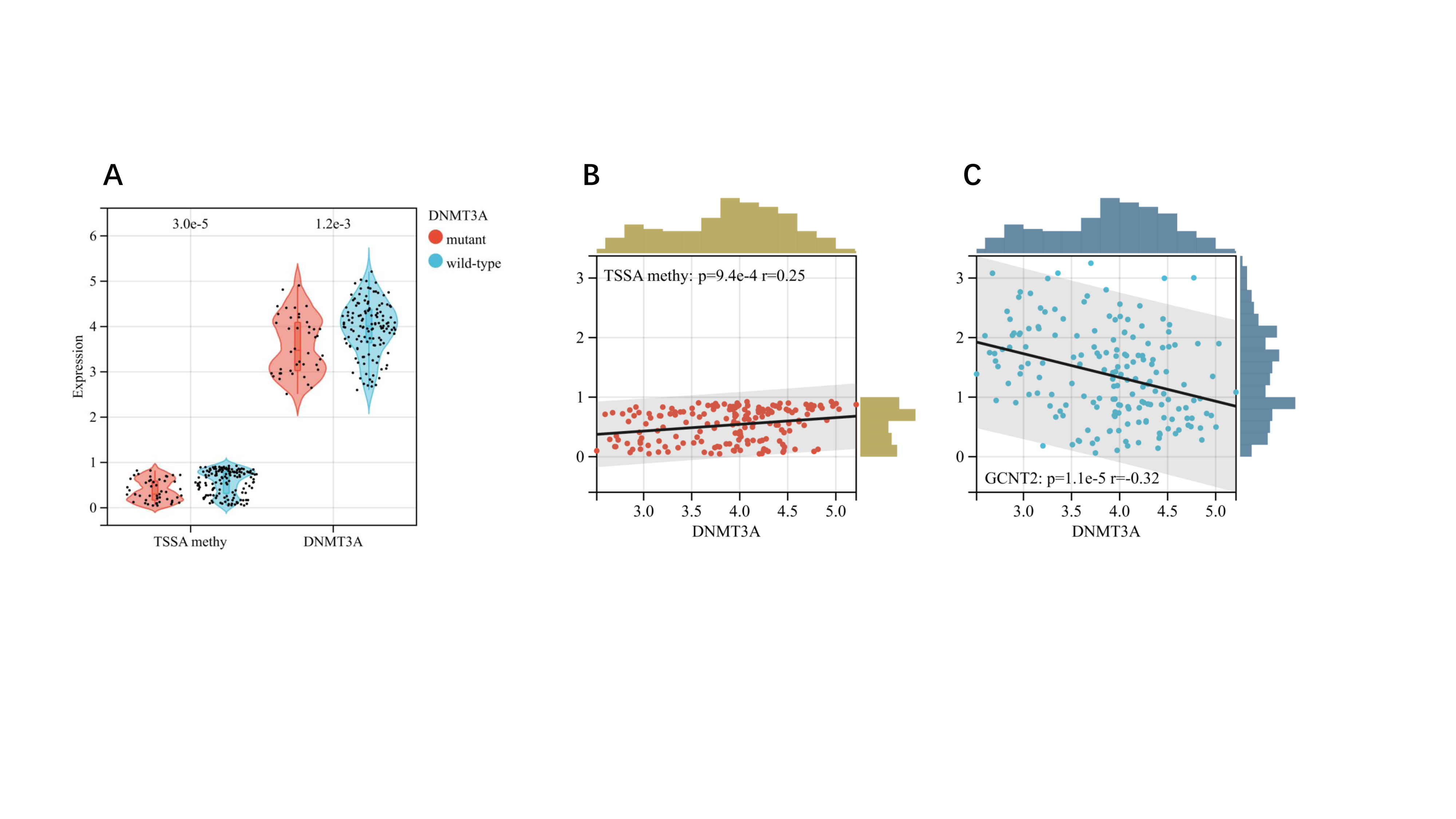

Supplement: Supplementary file 2 [file Image2.tif]
